# Zygospore development of *Spirogyra* (Charophyta) investigated by serial block-face scanning electron microscopy and 3D reconstructions

**DOI:** 10.3389/fpls.2024.1358974

**Published:** 2024-03-14

**Authors:** Sebastian J. Antreich, Charlotte Permann, Nannan Xiao, Giuseppe Tiloca, Andreas Holzinger

**Affiliations:** ^1^ Department of Bionanosciences, University of Natural Resource and Life Sciences, Vienna, Austria; ^2^ Department of Botany, University of Innsbruck, Innsbruck, Austria

**Keywords:** barite (BaSO_4_), cell wall, conjugation, helicoidal microfibrils, lipid droplets, sporopollenin, sexual reproduction, starch degradation

## Abstract

Sexual reproduction of Zygnematophyceae by conjugation is a less investigated topic due to the difficulties of the induction of this process and zygospore ripening under laboratory conditions. For this study, we collected field sampled zygospores of *Spirogyra mirabilis* and three additional *Spirogyra* strains in Austria and Greece. Serial block-face scanning electron microscopy was performed on high pressure frozen and freeze substituted zygospores and 3D reconstructions were generated, allowing a comprehensive insight into the process of zygospore maturation, involving storage compound and organelle rearrangements. Chloroplasts are drastically changed, while young stages contain both parental chloroplasts, the male chloroplasts are aborted and reorganised as ‘secondary vacuoles’ which initially contain plastoglobules and remnants of thylakoid membranes. The originally large pyrenoids and the volume of starch granules is significantly reduced during maturation (young: 8 ± 5 µm³, mature: 0.2 ± 0.2 µm³). In contrast, lipid droplets (LDs) increase significantly in number upon zygospore maturation, while simultaneously getting smaller (young: 21 ± 18 µm³, mature: 0.1 ± 0.2 and 0.5 ± 0.9 µm³). Only in *S. mirabilis* the LD volume increases (34 ± 29 µm³), occupying ~50% of the zygospore volume. Mature zygospores contain barite crystals as confirmed by Raman spectroscopy with a size of 0.02 - 0.05 µm³. The initially thin zygospore cell wall (~0.5 µm endospore, ~0.8 µm exospore) increases in thickness and develops a distinct, electron dense mesospore, which has a reticulate appearance (~1.4 µm) in *Spirogyra* sp. from Greece. The exo- and endospore show cellulose microfibrils in a helicoidal pattern. In the denser endospore, pitch angles of the microfibril layers were calculated: ~18 ± 3° in *S. mirabilis*, ~20 ± 3° in *Spirogyra* sp. from Austria and ~38 ± 8° in *Spirogyra* sp. from Greece. Overall this study gives new insights into *Spirogyra* sp. zygospore development, crucial for survival during dry periods and dispersal of this genus.

## Introduction

Sexual reproduction and the formation of resistant propagules in the form of spores or zygospores represent crucial parts in the life cycle of many plants, including basal groups like bryophytes or algae. Within the latter, especially streptophyte green algae have been in the centre of recent evolutionary studies as well as their adaptation strategies to abiotic stress factors. Streptophyte algae are divided into two major groups termed ‘KCM-grade’ (Klebsormidiophyceae, Chlorokybophyceae and Mesostigmatophyceae) and ‘ZCC-grade’ (Zygnematophyceae, Coleochaetophyceae and Charophyceae) (de Vries et al., 2016). Evolutionary, the ‘ZCC grade’ has been established as higher branching and the encompassed class Zygnematopyhceae as immediate sister lineage to land plants. This unique phylogenetic placement has sparked great interest in the phylogenomic features of this class, aiding the effort to elucidate the character state of the shared common algal ancestor of Embryophyta and streptophyte algae. Another focus has been set on their unique way of sexual reproduction. Interestingly, sexual reproduction has not been reported from members of the lower branching ‘KCM-grade’, but is well documented in Coleochaetophyceae (Domozych et al., 2016), Charophyceae (Nishiyama et al., 2018) and Zygnematophyceae (Kadlubowska, 1984). However, Zygnematophyceae are the only class in which sexual reproduction is performed without flagellated stages strictly by conjugation. This observation in conjunction with this class’s phylogenetic placement leads to the assumption that conjugation was an important factor in the evolution of land plants and the conquest of land. Zygnematopyhceae indeed often inhabit semi-terrestrial habitats, where they are exposed to increased abiotic stresses. Leaving the protective surroundings of perennial water bodies entails higher levels of UV-radiation, more drastic temperature shifts as well as desiccation stress. While dividing vegetative cells are indispensable in the persistence and expansion of populations, such often periodically occurring harsh environmental conditions necessitate the production of specialised resting stages. For example, in *Zygnema* sp. vegetative pre-akinetes are formed, which are characterised by the accumulation of storage compounds like lipid droplets (LDs) and the formation of thick cell walls making them resistant against different abiotic stresses (Holzinger and Pichrtová, 2016; Pichrtová et al., 2016). While it is not clear if zygnematophyceaen LDs are homologous in their molecular makeup to land plants, oleosin transcripts are drastically upregulated upon heat or desiccation stress (Rippin et al., 2017; de Vries and Ischebeck, 2020; de Vries et al., 2020; Dadras et al., 2023).

Zygospores are the product of sexual reproduction in Zygnematophyceae, showing remarkable protection against environmental stresses by the formation of rigid, multilayered zygospore cell walls (Kadlubowska, 1984; Permann et al., 2021a, 2021b, 2022, 2023). These zygospore cell walls are typically three layered, with a cellulosic endo- and exospore and a mesospsore containing sporopollenin-like material (Poulíčková et al., 2007). Recently, new insights on zygospore composition have been gained by Raman spectroscopy in *Mougeotia* sp. and *Spirogyra* sp. zygospores (Permann et al., 2021a; 2021b, 2022). Moreover, three-dimensional reconstruction of *Zygnema* sp. zygospores after focussed ion beam-scanning electron microscopy (FIB SEM) analysis allowed the analysis of the development of the zygospore cell walls and internal reorganisations during zygospore development (Permann et al., 2023). While in this study the main focus was on starch degradation and lipid rearrangement, no information on vacuole organisation is given (Permann et al., 2023).

For the present study we use *Spirogyra* spp. zygospores from field samples collected from different locations in Tyrol and Styria (Austria) and Greece. One strain, *Spirogyra mirabilis* has been previously determined and phylogenetically characterised (Permann et al., 2021a), the other strains were not phylogenetically analysed. We hypothesise a rearrangement of vacuoles and a drastic change of storage compounds during the maturation process in the form of degrading starch granules and size and volume increase of LDs. Particularly starch will be converted into building blocks for cell wall development, leading to the formation of the endo- and exospore, while lipids are converted to the aromatic compounds of the mesospore as previously described in *Spirogyra* sp. (Permann et al., 2021a; 2022). It will be interesting to analyse if these layers of the zygospore walls are of comparable size and texture in the different samples, which will be quantified for the first time after serial block-face scanning electron microscopy (SBF SEM). Barite crystals have been described in vegetative samples of *Spirogyra* sp. (Kreger and Boere, 1969; Barbosa et al., 2022), thus we hypothesise that barite crystals are also present in *Spirogyra* sp. zygospores. Despite several light- and electron microscopical observations of the conjugation process of *Spirogyra* sp. (Chmielevsky, 1890; Tröndle, 1907; Fowke and Pickett-Heaps, 1971; Ogawa, 1982; Takano et al., 2019; Permann et al., 2021a) and data on the cell wall composition of their zygospores has become available recently (Permann et al., 2022; Pfeifer et al., 2022), nothing is known on their 3D architecture and no quantitative data on changes during the maturation process are available. A better insight into these processes will contribute to the knowledge on maturation processes in zygospores and further our understanding of the success of Zygnematophyceae in conquering land.

## Material and methods

### Algal samples

Zygospores and filaments from different *Spirogyra* spp., derived from Austria (Tyrol, Styria) and Greece. In Austria, zygospores of *Spirogyra* sp. ‘Tyrol’ were collected in a puddle at 2250 m a.s.l. above the village Kühtai (Tyrol, Austria; 47°13’14.2”N, 11°01’24.6”E, Permann et al., 2022), *Spirogyra mirabilis* was sampled from a small rivulet near the main road close to Kühtai at 2020 m a.s.l. (Tyrol, Austria; 47°13’08.1’’N, 11°02’21.5’’E; Permann et al., 2021a) and *Spirogyra* sp. ‘Styria’ zygospores were collected in a small streamlet in the village Unterausersbach (46°51’56.5”N, 15°46’11.8”E) in Styria. Moreover, *Spirogyra* sp. ‘Greece’ zygospores were collected in a spring close to an ancient Castle of Karya (37°37’54.1”N, 22°32’25.8”E, 952 m a.s.l.) in Greece.

### Light-, transmission- and scanning electron microscopy

Fresh zygospore samples were viewed with an Axiovert 200 M inverted light microscope (Carl Zeiss AG, Jena, Germany), equipped with a MRc5 camera (Carl Zeiss AG, Jena, Germany).

For transmission electron microscopy (TEM) and serial block-face scanning electron microscopy (SBF SEM) samples were high pressure frozen (HPF) with a LEICA EMPACT high pressure freezer and freeze substituted (FS) in a Leica EM AFS FS apparatus (Leica Microsystems GmbH, Vienna, Austria) in 2% osmiumtetroxide (OsO_4_) and 0.05% Uranyl-acetat in acetone at -80°C for 60h, temperature was then raised to -30°C within 5h (10°C h^-1^), kept at -30°C for 4h and temperature finally raised to 20°C within 20h (2.5°C h^-1^) according to Aichinger and Lütz-Meindl (2005). Samples were embedded in Agar Low viscosity resin kit (Agar Scientific, Essex, UK).

Transmission electron microscopy of ultrathin sections, stained with 2% uranyl acetate and Reynold’s lead citrate was performed on a Zeiss Libra 120 transmission electron microscope (Carl Zeiss AG, Oberkochen Germany) at 80 kV, which was equipped with a TRS 2k SSCCD camera and operated by ImageSP software (Albert Tröndle Restlichtverstärker Systeme, Moorenweis, Germany).

Scanning electron microscopy was performed with an Apreo SEM (Thermofischer Scientific Inc., MA USA) at 1 kV and 100 pA.

### Serial block-face scanning electron microscopy (SBF SEM) and segmentation

Embedded samples were cut into small pieces and glued with two-component silver cement on aluminium stubs (1h at 90°C). Stubs were then transferred to the Ultracut (UC7, Leica Microsystems GmbH, Vienna, Austria) and the glued resin block was further trimmed with a glass knife to a cube of 0.5 mm edge length following the protocol of Antreich et al. (2021). Thereafter, the stub was put into the Apreo SEM with an inbuilt microtome equipped with a diamond knife. Serial cuts of 100 nm depth were performed until a whole zygospore was scanned. Resolution was set to 10 nm per pixel and each surface was scanned with 1.18 kV and 100 pA.

Picture stacks were scaled (40 nm per pixel) and aligned with the software ImageJ (Rueden et al., 2017) using the linear stack alignment plugin (Lowe, 2004). Aligned stacks were transferred to the Amira software (Avizo, Thermofischer Scientific Inc., MA, USA) for segmentation. Each zygospore was segmented into cell wall, LDs, chloroplasts with pyrenoids and starch, vacuoles with crystals, and nucleus.

### Helicoidal microfibril pitch angle calculation

TEM images of the endospore of *Spirogyra mirabilis*, *Spirogyra* sp. ‘Tyrol’ and *Spirogyra* sp. ‘Greece’ were used to measure the average distance between a 180° turn of the microfibril layers with ImageJ, by analysing the differences in grey values caused by the rotations. The diameter of the individual microfibrils was estimated by analysing scanning electron micrographs of cracked surfaces of freeze dried zygospores. To calculate the pitch angle of the microfibirl layers, the average length of a 180° turn of the layers was divided by the average thickness of the microfibrils according to Xiao et al. (2021). 3D reconstructions of the helicoidal cellulose fibril patterns were performed with FreeCAD (https://www.freecad.org).

### Raman spectroscopy

Fresh samples from Styria (Austria) and Greece were put on glass slides and analysed under a confocal Raman microscope (Alpha300RA, WITec GmbH), as previously described by Permann et al. (2021b). The samples were scanned with a laser power (excitation wavelength of 532 nm) of 20mW and the integration time was set to 0.1s. Project FIVE Plus (WITec GmbH, Germany) with the integrated True Component Analysis Tool used for spectral processing and data analysis. After cosmic ray removal and baseline correction, different peak positions were integrated to display the chemical distribution maps.

### Statistical analysis

For statistical evaluation of the starch, LDs and barite crystal volumes, the software STATISTICA 7.1 (StatSoft.) was used. A Kruskal–Wallis one-way ANOVA on ranks was applied. followed by multiple comparisons of the mean group ranks. Significant differences (*p* < 0.05) are indicated by lower case letters.

## Results

### Light microscopy shows differences in the investigated samples

**Figure 1:**
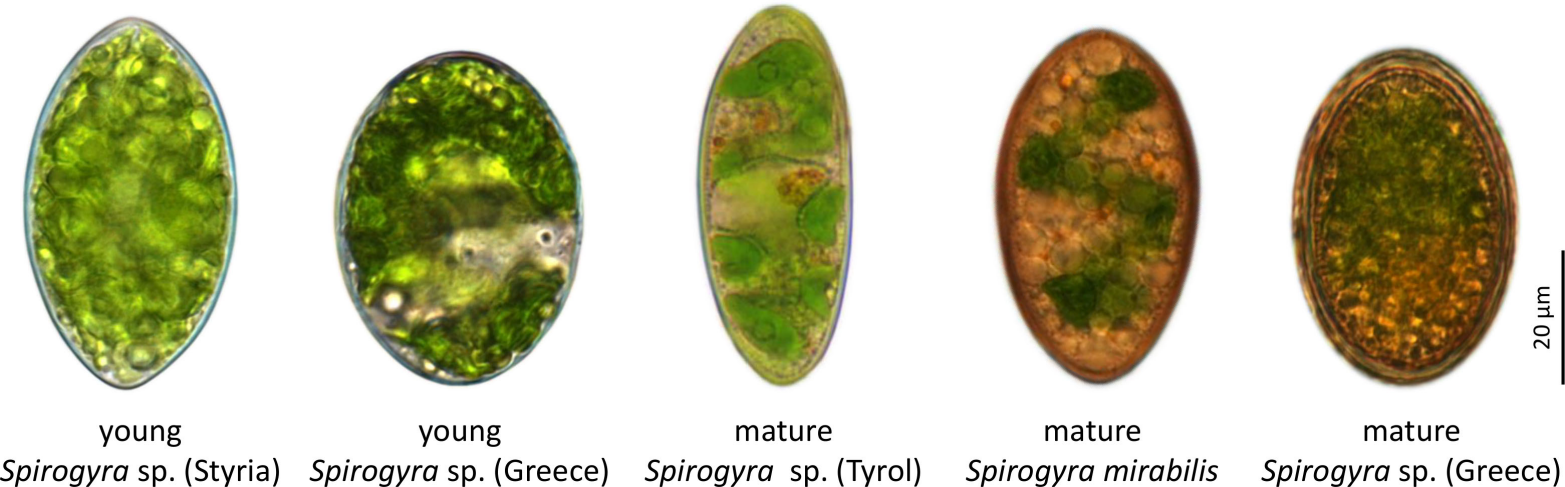
Light micrographs of investigated *Spirogyra* zygospores. With this scheme different developmental stages of the zygospores are described, while *Spirogyra* sp. ‘Styria’ and ‘Greece’ contained young stages, in *Spirogyra* sp. ‘Tyrol’, *S. mirabilis* and *Spirogyra* sp. ‘Greece’ mature developmental stages were detected.

In this study, we used *Spirogyra* sp. zygospores from four different locations, some of which were previously characterised, like *S. mirabilis* (Permann et al., 2021a) or *Spirogyra* sp. ‘Tyrol’ (Permann et al., 2022), while other samples, like *Spirogyra* sp. ‘Styria’ and ‘Greece’ are used in this study for the first time (**Figure 1**). Despite the conjugation and zygospore morphology being the classical taxonomic trait (Kadlubowska, 1984; Stancheva et al., 2013; Takano et al., 2019), it is difficult and not the aim of this study to perform a taxonomic or even phylogenetic analysis of the collected samples, and we use only the collection localities to describe the samples.

### Serial block face scanning electron microscopy gives new insights into the 3D structure

**Figure 2:**
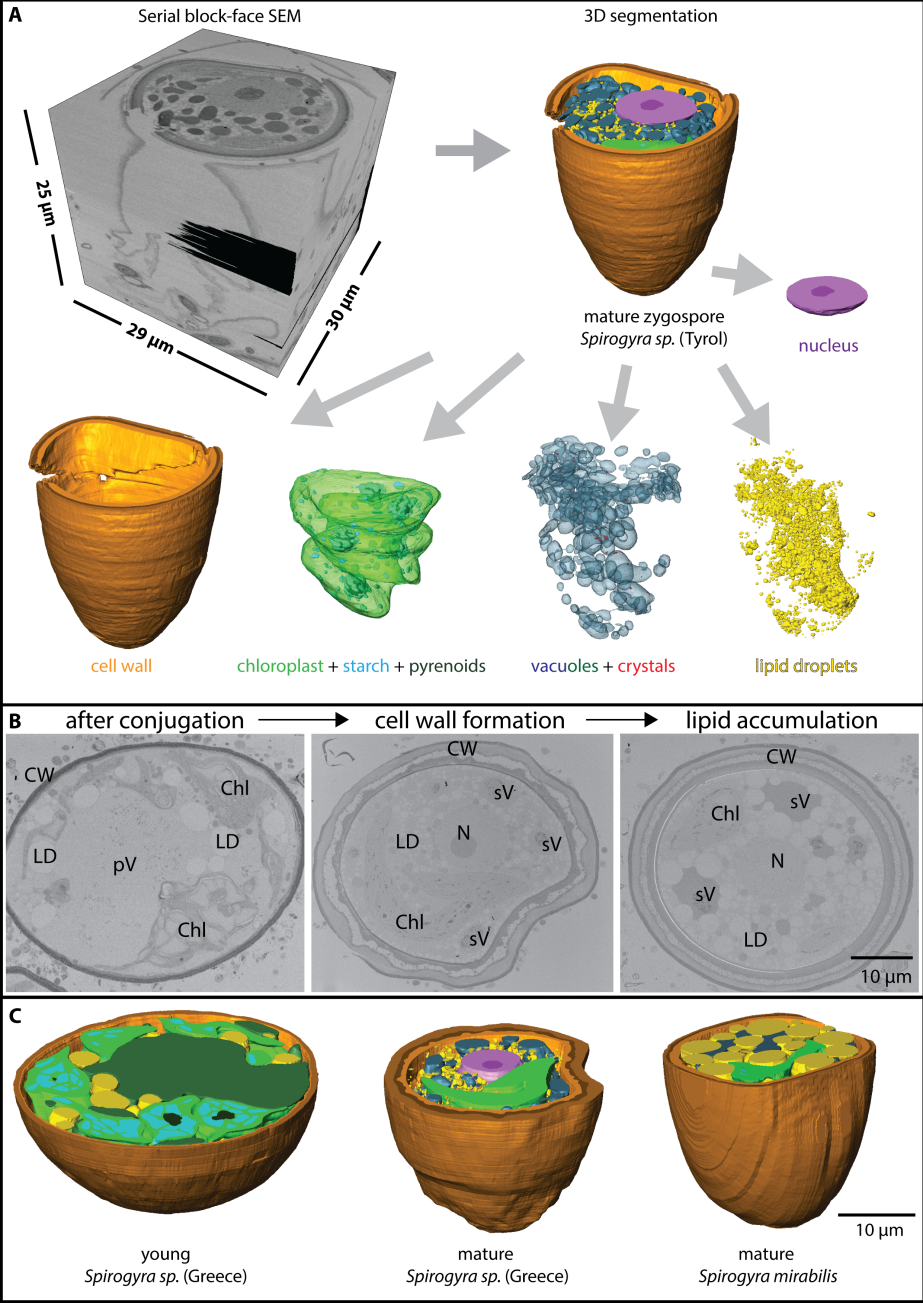
**(A)** Process of serial block face scanning electron microscopy with stack generation (top left) and 3D segmentation (top right) in *Spirogyra* sp. ‘Tyrol’, the segments can be extracted and are separately shown (bottom), **(B)** 2D view of *Spirogyra* sp. ‘ Greece’ in different developmental stages, shortly after conjugation (left), during zygospore wall formation (middle) and lipid accumulation (right), cellular structures depicted are pV ‘primary vacuoles’, sV ‘secondary vacuoles’, LD lipid droplets, Chl chloroplasts, N nucleus and CW cell wall, **(C)** 3D reconstruction of young and mature zygospore segments of different *Spirogyra* sp.

As the SBF SEM process is quite time consuming, only a limited number of samples could be analysed. Therefore, this paper is more focusing on the qualitative changes during *Spirogyra* zygospore formation. Still some quantitative aspects were included to highlight possible trends. Reconstructions generated by SBF SEM gave a better understanding of the organelle shapes and their distribution within the cell (**Figure 2A**). The individual organelles, in particular the chloroplasts with starch granules and pyrenoids, vacuoles with crystals, LDs, as well as cell wall layers were successfully separated and the corresponding 3D reconstructions were analysed.

### Zygospore maturation process

The whole process of zygospore maturation was shown exemplarily in the series of *Spirogyra* sp. ‘Greece’ in 2D (**Figure 2B**) and also by means of 3D reconstructions of different maturation stages from different *Spirogyra* sp. strains (‘Greece’ and *S. mirabilis*, **Figure 2C**). After conjugation, during early zygospore formation, cell structure changed drastically. A young zygospore of *Spirogyra* sp. ‘Greece’ still contained two nuclei (**Supplementary Figure 1**) and two spiral chloroplasts with multiple pyrenoids surrounded by large starch granules, giving the chloroplast the appearance of a pearl string (**Figure 2B**). The ‘primary vacuoles’, originating from the vacuoles of the gametes became more fragmented and were lacking crystals (**Figure 2B**, left). In contrast, the vacuoles which were fragmented all over the cell lumen into different sized compartments and contained many crystals and plastoglobules were termed ‘secondary vacuoles’ (**Figure 2B**, middle/right). Zygospores showed either multiple small LDs (**Figure 2C**, middle) or a few large ones all around the lumen (**Figure 2C**, right). During progressing zygospore development, the cell wall became thicker and three layered (**Figures 2B, C**).

### Chloroplasts change in shape while starch granules degrade

**Figure 3:**
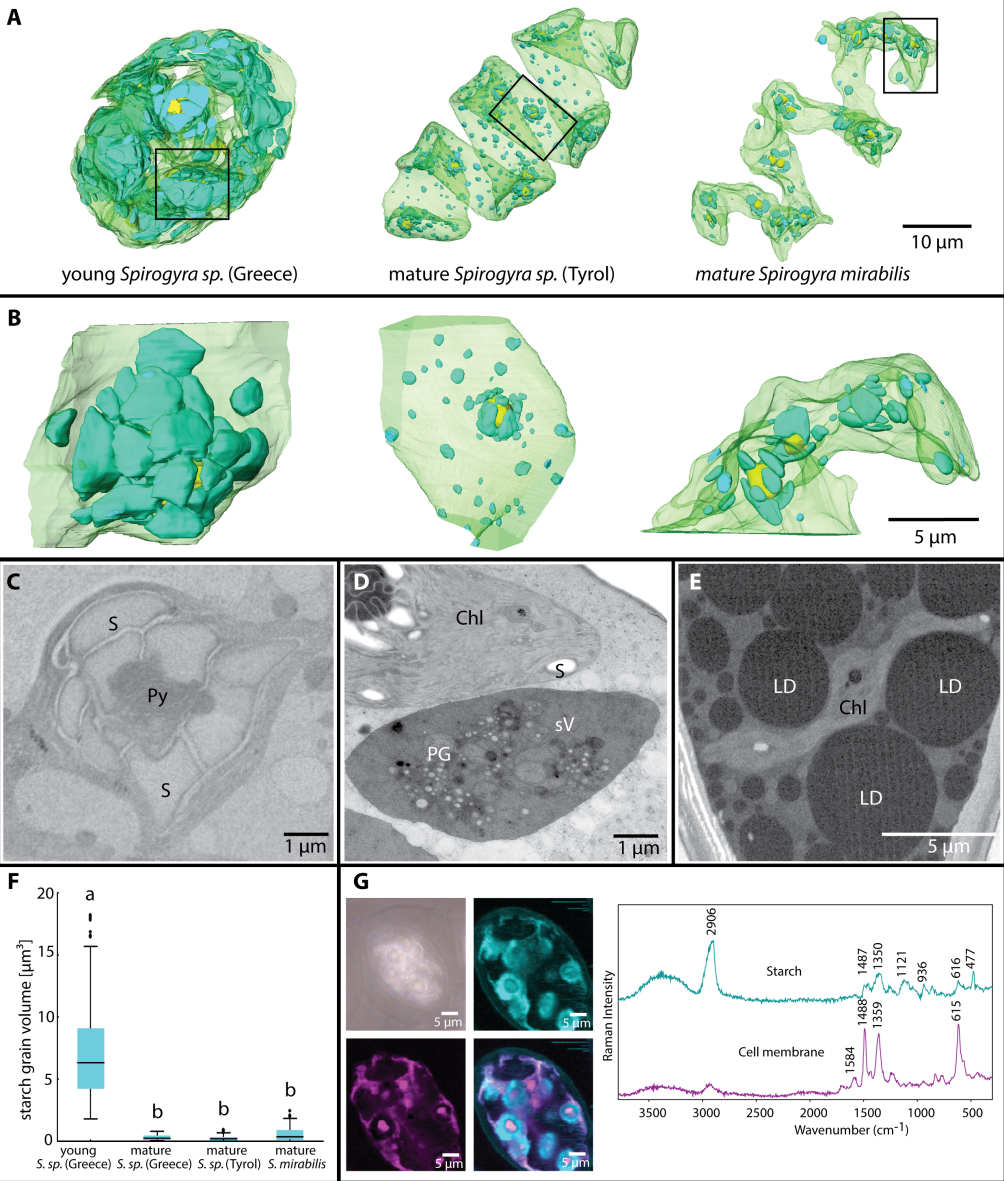
**(A)** Chloroplast shapes in young *Spirogyra* sp. ‘Greece’, containing all parental chloroplasts leading to a very dense appearance, in contrast, mature zygospores of different *Spirogyra* sp. show one spiral chloroplast, **(B)** small segments from **(A)** illustrating the high density of large starch granules in the young versus the mature *Spirogyra* sp. zygospores, **(C)** 2D view of young *Spirogyra* sp. ‘Greece’ chloroplast with pyrenoid (Py) surrounded by numerous starch granules, **(D)** 2D view of mature *Spirogyra* sp. ‘Tyrol’ showing a part of the intact chloroplast (Chl) with starch granules (S) and the degraded chloroplast leading to the formation of the secondary vacuole (sV), still containing plastoglobules (PG), **(E)** 2D view of mature *Spirogyra mirabilis* with large lipid droplets (LDs) and reduced chloroplast (chl), **(F)** analysis of the starch volume distribution at different developmental stages, **(G)** Raman imaging based on “true component analysis” reveal starch and cell wall components in a living zygospore of *Spirogyra* sp. ‘Styria’.

The chloroplasts of young and mature *Spirogyra* zygospores showed a spiral configuration, however, their shape changed depending on the starch within or the other organelles around the chloroplast (**Figures 3A, B**). The appearance of chloroplasts drastically changed after conjugation, where the two chloroplasts of the male and female gametes were stronger coiled within the zygospore and contained many pyrenoids and massive starch granules surrounding them (**Figures 3B**, left, **C**). The chloroplasts filled up a large portion of the zygospore lumen (**Figure 3A**, left), as also illustrated in fresh samples of *Spirogyra* sp. ‘Styria’ and ‘Greece’ (**Figure 1**). The chloroplasts of the male gamete degraded into an electron dense secondary vacuole, which was clearly recognizable by remaining thylakoid membrane fragments as well as plastoglobules (**Figure 3D**). As the zygospore matured, chloroplasts showed a distinct spiral shape (**Figure 3A**). However, we also observed cases (*Spirogyra* sp. ‘Tyrol’) where the spiral was fragmented into four single sections (**Supplementary Figure 2**). The pyrenoids and starch granules were still present, but much less and smaller when compared with the young zygospore after conjugation (**Figure 3B**). In the case of mature *S. mirabilis*, the shape was still spiral-like but the large LDs formed multiple indentations in the chloroplast (**Figure 3E**). The starch degradation was quantified by the 3D reconstructions, where young *Spirogyra* sp. ‘Greece’ had a significantly (*p* < 0.05) higher volume of 8 ± 5 µm³ when compared with mature zygospores of the same strain (0.2 ± 0.2 µm³) and *S. mirabilis* (0.6 ± 0.6 µm³) or *Spirogyra* sp. ‘Tyrol’ (0.3 ± 0.2 µm³), which did not differ significantly (**Figure 3F**). Raman analysis confirmed the presence of the starch granules (marker bands 477, 936, 1121, 1350, and 2906 cm^-1^) around the pyrenoids and illustrated cellular membranes in freshly collected young zygospores of *Spirogyra* sp. ‘Styria’ (**Figure 3G**).

### High variability in LDs size and abundance during zygospore maturation

**Figure 4:**
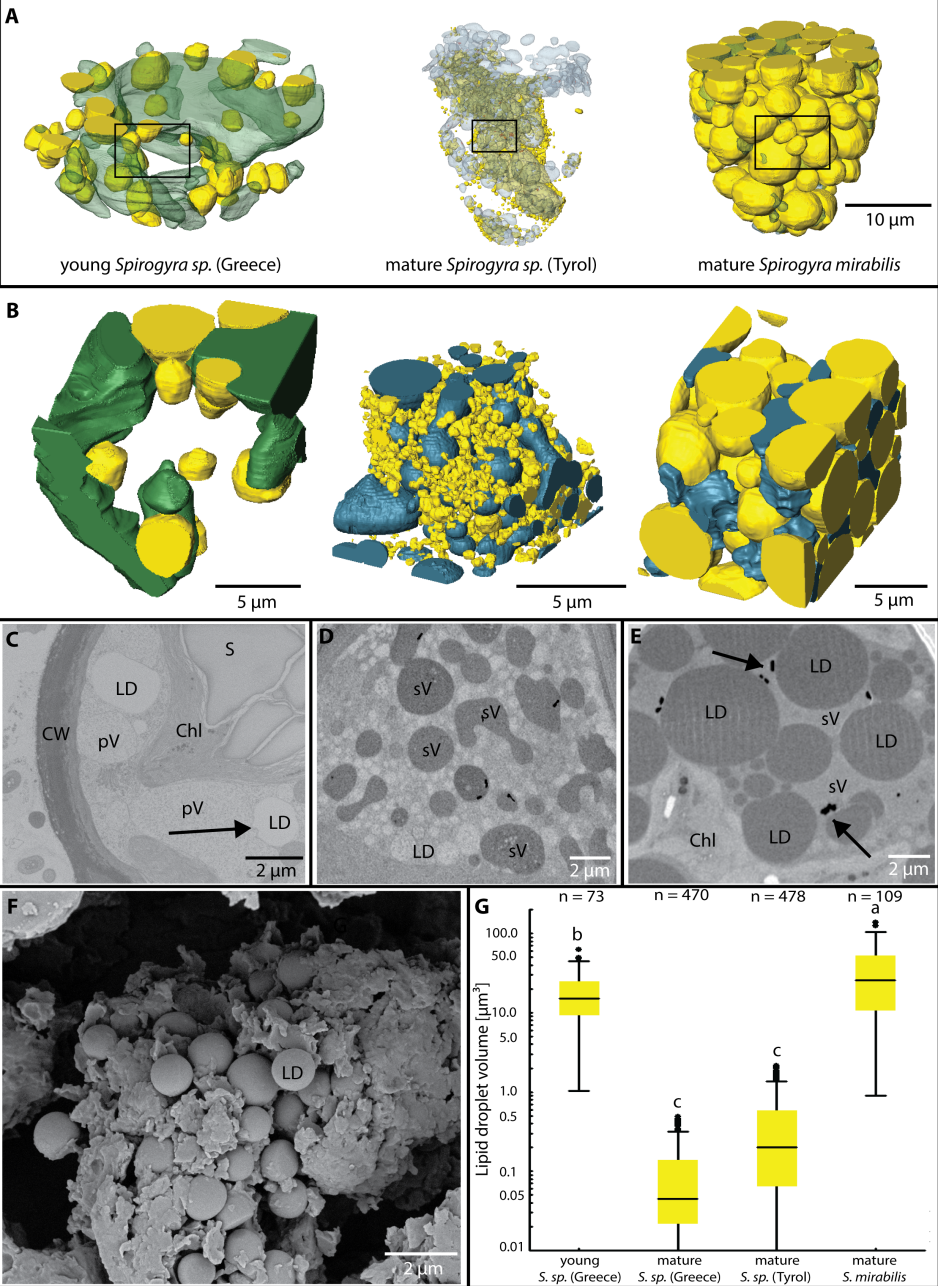
**(A)** Lipid droplets (LDs) and vacuoles in different *Spirogyra* sp. zygospores, **(B)** small segments from **(A)** illustrating the relationship between LDs and the vacuoles, note that larger LDs in *S. mirabilis* reduce the space for vacuoles, **(C)** 2D view of young *Spirogyra* sp. ‘Greece’, illustrating ‘primary vacuoles’ (pV) and the close contact to LDs (arrow), chloroplast (Chl) intact, containing large starch granules (S), **(D)** 2D view of mature *Spirogyra* sp. ‘Tyrol’ illustrating ‘secondary vacuoles’ (sV) and numerous less electron dense LDs, **(E)** large LDs in mature *S. mirabilis* reducing the volume of ‘secondary vacuoles’ containing barite crystals (arrows), **(F)** scanning electron micrograph of mature *Spirogyra* sp. ‘Greece’ shows many LDs, **(G)** LD volumes of different *Spirogyra* sp., significant differences (*p* < 0.05) are indicated by different lower case letters.

Lipid droplets were present in all investigated *Spirogyra* (**Figures 4A, B**). In young zygospores of *Spirogyra* sp. ‘Greece’ after conjugation, the LDs were large (21 ± 18 µm³) and mostly connected to a vacuole with low electron density (**Figure 4C**). The LDs had a more irregular shape and the connection between them and the vacuoles were wavy. In the mature zygospores of *Spirogyra* sp. ‘Tyrol’ and ‘Greece’ the LDs were much smaller (0.1 ± 0.2 and 0.5 ± 0.9 µm³), but numerous (**Figures 4B, D, G**). The round shape of the LDs is illustrated in a scanning electron micrograph (**Figure 4F**). In contrast, in mature zygospores of *S. mirabilis*, the LDs were much larger (34 ± 29 µm³) and became electron dense (**Figures 3E**, **4E**). In *S. mirabilis*, LDs took up around half of the lumen of the zygospores and squeezed the other organelles like chloroplasts between them (**Figure 3E**). Box plots show the size class distribution of LDs, young *Spirogyra* sp. ‘Greece’ had significantly larger LDs than mature *Spirogyra* sp. ‘Greece’ or ‘Tyrol’ (*p* < 0.05, **Figure 4G**). In contrast, in mature *S. mirabilis* the LDs were significantly larger when compared to the other investigated zygospores (*p* < 0.05, **Figure 4G**).

### Vacuoles get fragmented and enriched with barite crystals

**Figure 5:**
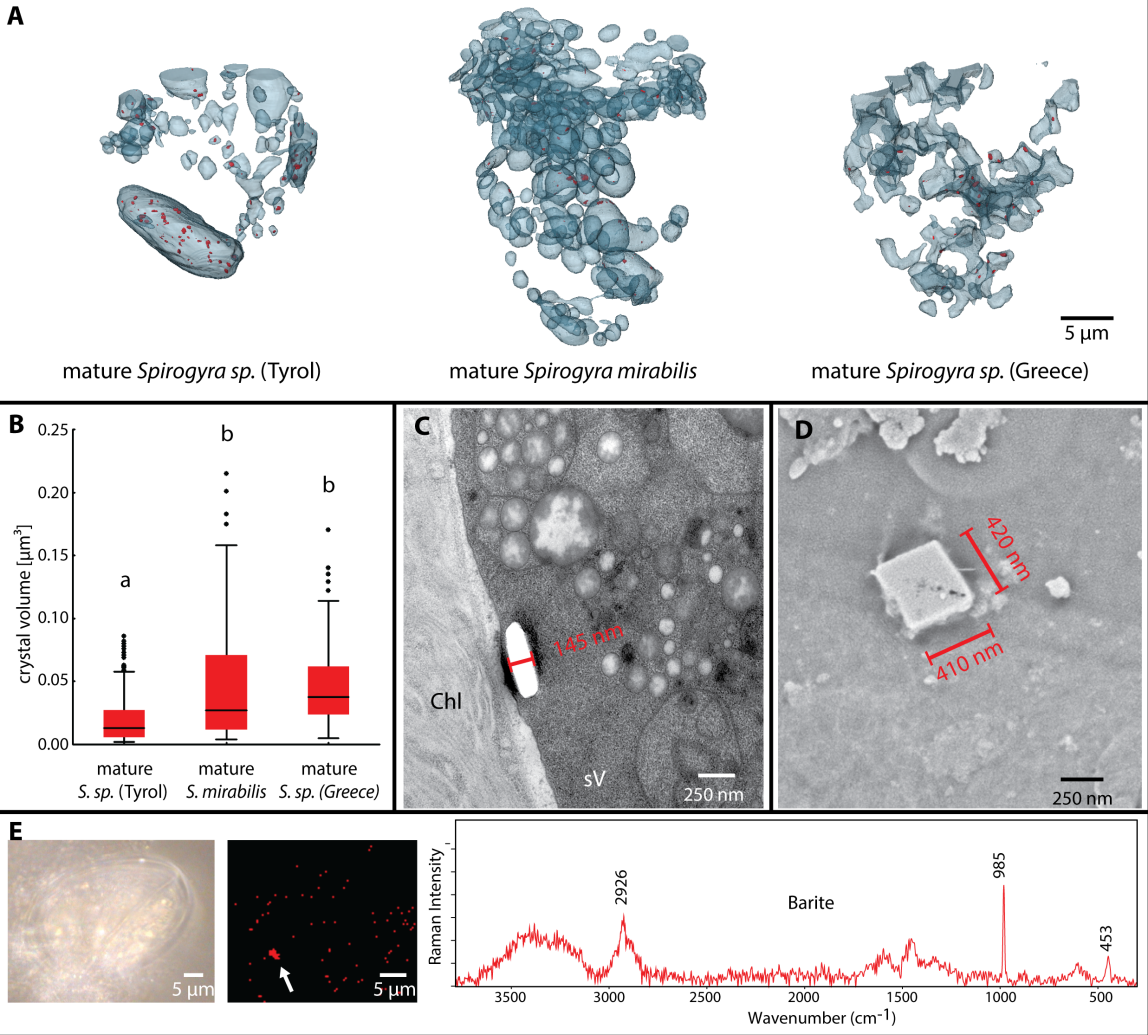
Barite crystals in mature *Spirogyra* sp. zygospores. **(A)** distribution of barite crystals (red) in vacuoles of different strains, **(B)** quantification of crystal sizes, **(C)** TEM of crystal (broken out) in secondary vacuole of *Spirogyra* sp. ‘Tyrol’, **(D)** scanning electron micrograph of crystal in *Spirogyra* sp. ‘Greece’, **(E)** Raman spectroscopy and barite detection in zygospores of *Spirogyra* sp. ‘Greece’.

In young zygospores of *Spirogyra* sp. ‘Greece’, ‘primary vacuoles’ started to fragment (**Figures 4A, B**, left). In the analysed samples no crystals were visible in these vacuoles. The mature zygospores of *Spirogyra* sp. ‘Tyrol’, ‘Greece’ and *S. mirabilis* contained several fragmented ‘secondary vacuoles’ (**Figure 5A**), which contained many crystals in a size range of 0.02 - 0.05 µm³, significantly smaller in *Spirogyra* sp. ‘Tyrol’, (*p* < 0.05, **Figure 5B**). Besides the crystals, multiple vacuoles contained dark stained plastoglobules, suggesting their origin as direct remnants of the abandoned chloroplasts (**Figure 5C**). Transmission electron micrographs (**Figure 5C**) and scanning electron micrographs (**Figure 5D**) showed the shape of the crystals. Additionally, Raman spectra confirmed that the crystals were composed of barite shown by the marker bands at 453 nm and 985 nm (**Figure 5E**).

### Cell walls of zygospores show differences in layering

**Figure 6:**
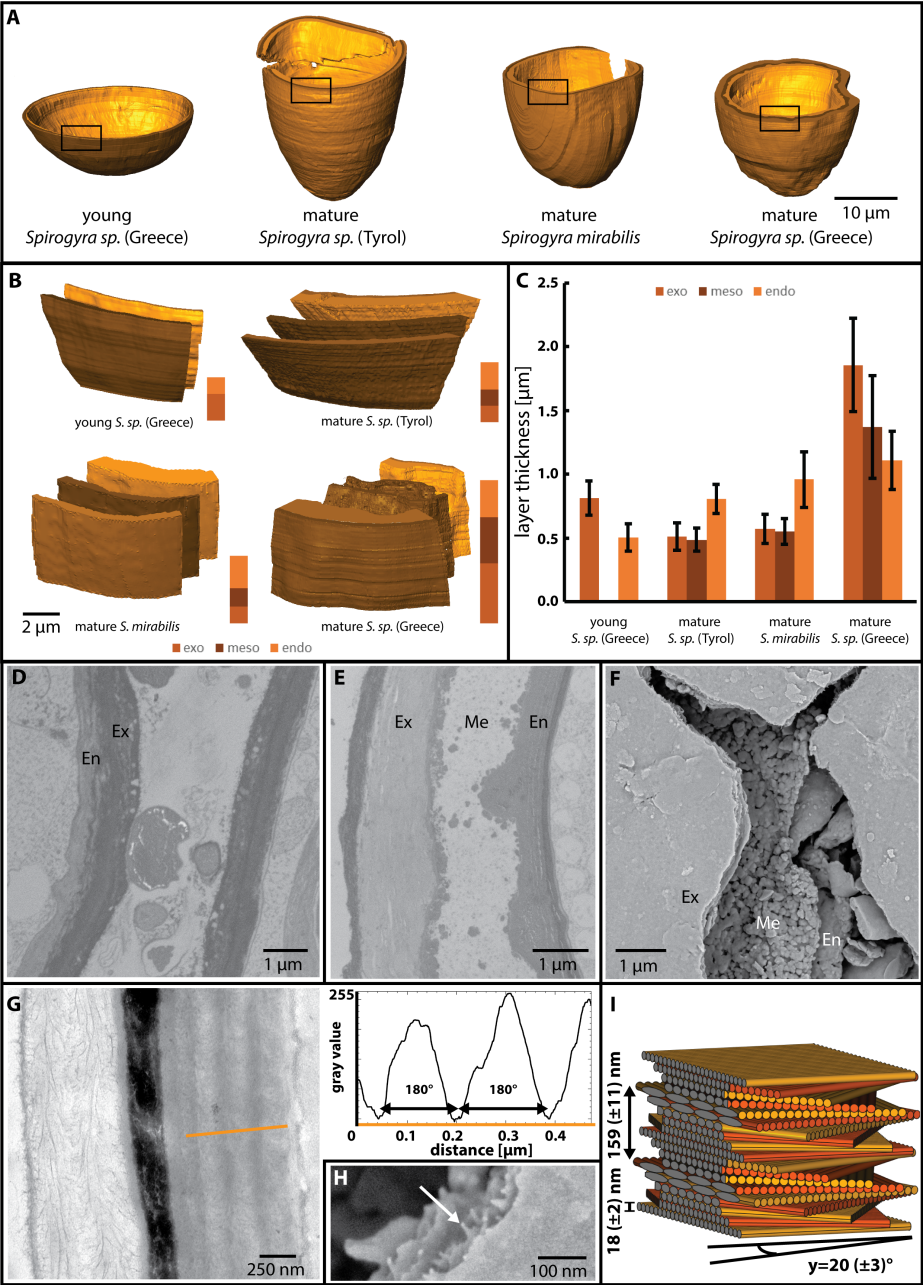
Cell wall features of different *Spirogyra* sp. as indicated. **(D-F, H)** *Spirogyra* sp. ‘Greece’, G, **(I)** *Spirogyra* sp. ‘Tyrol’. **(A)** reconstruction of young and mature zygospore cell walls, **(B)** visualisation of the different zygospore wall layers (exo-, meso- and endospore), **(C)** quantitative analysis of the layers sizes, showing mean values and standard deviations as derived from the reconstructions illustrated in **(A)**, **(D)** 2D view of young zygospore walls, only endospore (En) and exospore (Ex) are present, **(E)** 2D view of mature zygospore wall, notice the reticulate shape of the mesospore (Me), **(F)** scanning electron micrograph of cracked zygospore wall, **(G)** TEM of cell wall cross section indicates the area of measurement (orange line) for the grey scale determination of different layers of the endospore, **(H)** scanning electron micrograph illustrates individual cellulose microfibrils (arrow), **(I)** 3D reconstruction of the helicoidal pattern of the cellulose microfibrils.

Cell wall formation of the zygospores started shortly after conjugation and was reconstructed for all mature samples (**Figure 6A**). In young zygospores of *Spirogyra* sp. ‘Greece’ after conjugation, the cell wall increased in thickness and became clearly two layered (**Figure 6B**). The outer layer (exospore) was electron denser and thicker than the inner layer (endospore) (**Figure 6D**). The mature zygospores of *Spirogyra* sp. ‘Tyrol’, *S. mirabilis* and *Spirogyra* sp. ‘Greece’ revealed thick, multi-layered cell walls (**Figures 6B, C**) and their thickness ranged between 1.8 and 4.2 µm in average (**Figure 6C**). In all cases, the endospore was composed of a thin electron dense layer towards the lumen and a thicker layer composed of helicoidal arranged cellulose fibres (see below). The mesospore showed the strongest variety between the individual *Spirogyra* species (**Figures 6B, C**). In *Spirogyra* sp. ‘Greece’, the mesospore was electron dense only at the edges, but electron translucent in the centre, which suggests a reticulate structure (**Figures 6E, F**). In the other *Spirogyra* species, the cell wall showed a different arrangement with an electron dense but unstructured mesospore (**Figures 6B, C**). The exospore was loosely layered cellulose and exhibited an electron dense layer towards the outside (**Figure 6G**).

### Helicoidal pattern of the cellulose microfibres

The exo- and endospore showed cellulose microfibrils in a helicoidal pattern and an analysis of the pitch angle of the microfibril layers in the endospore was performed. The grey values were measured (orange line, **Figure 6G**) and subsequently the distances between the minima were determined (**Figure 6G**, graph). The diameter of the individual cellulose fibrils was estimated in SEM micrographs (**Figure 6H**). The following pitch angles were calculated: ~18 ± 3° in *S. mirabilis*, ~20 ± 3° in *Spirogyra* sp. ‘Tyrol’ and ~38 ± 8° in *Spirogyra* sp. ‘Greece’. A 3D reconstruction of the microfibril arrangement in *Spirogyra* sp. ‘Tyrol’ is shown (**Figure 6I**).

## Discussion

*Spirogyra* zygospores at different maturation stages from different field samples were used to investigate the ultrastructural rearrangements during the entire process of zygospore formation. Due to the nature of field samples not all stages of zygospore development were present in each *Spirogyra* sample. However, by comparing different species, a comprehensive overview of the process could be revealed and quantifications of certain compartments (starch, LDs, barite crystals) could be determined. Also, in cases where the same stage was present, differences in the process were described, suggesting strain specific zygospore formation. These observations were complemented by a detailed analysis of the helicoidal pattern of the cellulose microfibrils in the endospore previously reported for *Spirogyra* sp. (Permann et al., 2022).

### Rearrangements during conjugation and zygospore formation

Conjugation and zygospore formation in *Spirogyra* have been studied by light- and transmission electron microscopy previously (e.g. Chmielevsky, 1890; Tröndle, 1907; Fowke and Pickett-Heaps, 1971; Ogawa, 1982; Permann et al., 2021a; 2022; Pfeifer et al., 2022), however, no attempts have been made to investigate this process by 3D reconstruction. It has been shown, that after the formation of papillae a fusion process between the male and female gametes is initiated, where the protoplast of the male gametes migrates into the female gametangium (Fowke and Pickett-Heaps, 1971; Permann et al., 2021a). This is a result of increased Golgi activity in the male filament (Fowke and Pickett-Heaps, 1971). Early zygospores still possess two nuclei (Fowke and Pickett-Heaps, 1971), as also found in the present study (**Supplementary Figure 1**), which form nuclear projections before they fuse (Ogawa, 1982). In later stages only one nucleus is found within a zygospore, as also shown by the recent study (**Figure 2A**).

### Chloroplasts undergo a drastic change during zygospore maturation

In all analysed stages of zygospore development, we found spiral chloroplasts, which is a strong characteristic of this species and has also been described for zygospores (Takano et al., 2019; Permann et al., 2021a; 2022).

In young developmental stages of the zygospores, right after conjugation, the spiral chloroplast appearance is hardest to observe, as the two chloroplasts derived from the male and female gametes are intertwined and have huge pyrenoid-starch complexes, which bulge the chloroplasts as observed in *Spirogyra* sp. ‘Greece’ (**Figures 3A, B**). Initial ultrastructural descriptions of the conjugation process in *Spirogyra* sp. were presented by Fowke and Pickett-Heaps (1971), illustrating this condition with tightly packed pyrenoids in the chloroplasts resulting from cytoplasmic condensation. The presence of these large complexes suggests a strong metabolic activity in young developing zygospores. High amounts of cell wall components and other metabolites need to be synthesised in *Spirogyra* (Permann et al., 2021a; 2022). However, during this process, the male chloroplast will be abandoned as already suggested by Chmielevsky (1890) and later described in detail by TEM, illustrating that 7 days after conjugation in *Spirogyra*, the male chloroplast was degraded by pre-vacuoles, but especially, the plastoglobules remained longer in the newly formed vacuoles (Ogawa, 1982). We observed a similar situation in *Spirogyra* sp. ‘Tyrol’ where the abandoned chloroplast got electron dense and contained plastoglobules and remnants of thylakoid membranes resulting in the formation of ‘secondary vacuoles’ (**Figure 3D**).

From the young zygospore to the mature stage, the remaining chloroplast gets reorganised into a clear spiral shape (**Figures 3A, B** *Spirogyra* sp. ‘Tyrol’ and *Spirogyra mirabilis*), however, in some cases it gets segmented into several parts. During the maturation process of *Spirogyra* zygospores, the most obvious change was that the pyrenoid-starch complexes got drastically smaller during zygospore maturation. While the average volume of starch granules was ~8 µm^3^ in young zygospores of *Spirogyra* sp. ‘Greece’, this value dropped to ~0.2 µm^3^ in mature zygospores of the same strain. This can be explained by the fact that many new building blocks for the massive zygospore cell wall are needed. A similar observation was made in *Zygnema vaginatum* zygospores, where the starch granule volume was reduced from 3.5 µm^3^ in young cells to 0.7 µm^3^ in an intermediate stage of zygospore formation (Permann et al., 2023). The nature of starch granules was moreover confirmed by confocal Raman spectroscopy, where the characteristic skeletal mode (ß(CCC)) at 480 cm^-1^ (Wiercigroch et al., 2017), in our case shifted to 477 cm^-1^ as well as other signature bands at 936, 1121, 1350 and 2906 cm^-1^ were detected, which were slightly shifted when compared to literature data (Wiercigroch et al., 2017).

### Changes in lipid droplets during zygospore maturation

Lipid droplets are storage products with a lipid ester core and a surface phospholipid monolayer, containing a special set of proteins shaping them (Huang, 2018). These proteins may differ drastically upon the phylogenetic position, while chlorophytes have a major lipid-droplet protein (MLDP), oleosins are typical for higher plants and were first reported in Charophytes (Huang et al., 2013). Interestingly, in *Spirogyra grevilleana*, oleosin transcript levels increased drastically in cells undergoing conjugation for zygote formation, and the LD fraction from these cells contained oleosins identified by proteomics (Huang et al., 2013).

After conjugation in young zygospores of *Spirogyra* sp. ‘Greece’ LDs are formed, which differ from the LDs found in the mature stage of this strain (**Figures 4A, B**). These LDs are strongly attached to vacuoles and are separated by a wavy interface (**Figure 4C**). Their mean volume was about 20 µm^3^, while in mature zygospores of *Spirogyra* sp. ‘Greece’ the mean volume was significantly reduced to ~0.1-0.5 µm^3^. For comparison, in *Zygnema vaginatum* young zygospores showed LDs with a variety of shapes and a broad size class distribution with an average volume of only 1.8 µm^3^ and a surface/volume ratio of 7.2 (Permann et al., 2023).

The LDs in the mature *Spirogyra* sp. ‘Tyrol’ and *S. mirabilis* are more spherical in shape and not connected to vacuoles. However, the size classes of LDs in mature zygospores varied drastically, in *Spirogyra* sp. ‘Tyrol’ their size was similarly reduced as in *Spirogyra* sp. ‘Greece’. In contrast, in *S. mirabilis* the mean volume of LDs was around 35 µm^3^ and up to 50% of the cell lumen were occupied by LDs. Such high levels of LDs are also found in stressed cells, reflecting a higher triacylglycerol (TAG) production in the plastid stroma (Ischebeck et al., 2020). Concluded from the higher electron density of LDs in *S. mirabilis* when compared with the other investigated strains, the composition of fatty acids could be different, containing more unsaturated fatty acids with a higher reactivity for osmium (Cheng et al., 2009). An analysis of LD composition has been performed in young and old (pre-akinete) *Zygnema* sp., suggesting that mainly two unsaturated fatty acids, oleic acid (C 18:1) and linoleic acid (C 18:2), were increased upon the maturation process (Pichrtová et al., 2016). Mature zygospores of *Zygnema vaginatum* had a layer-like distribution of LDs and they were found in direct contact with network-forming mitochondria in all developmental stages (Permann et al., 2023).

### Rearrangement of vacuoles in zygospores

While vegetative filaments of *Spirogyra* sp. possess large vacuoles, the conjugation process leads to a drastic reduction in the vacuolar volume (Fowke and Pickett-Heaps, 1971), which allows the newly formed zygospore to fit into the female gametangium. We termed them ‘primary vacuoles’, as they are remnants of the vacuoles of the gametes (**Figure 3C**). In contrast, the abortion of the male chloroplast leads to structures that we termed ‘secondary vacuoles’ (**Figures 3D, E**), and recently autophagy has emerged as an essential mechanism for selective degradation of the chloroplast (Zhuang and Jiang, 2019). Most interestingly, in the present study we could observe degradation products of the chloroplast in these ‘secondary vacuoles’, such as plastoglobules and remnants of thylakoid membranes. The detailed mechanisms remain to be elucidated, but a macroautophagy-like process is suggested for the selective degradation of the whole chloroplast (Zhuang and Jiang, 2019).

### Barite crystals in mature zygospores

While biomineralization of dolomite by *Spirogyra* sp. has been described recently (Del Buey and Sanz-Montero, 2023), several studies report on the occurrence of barite crystals in vegetative *Spirogyra* sp (Kreger and Boere, 1969; Barbosa et al., 2022). Ba^2+^ ions are usually toxic as they have the capacity to block K^+^ channels (Pilatová et al., 2023), but BaSO_4_ (barite) is insoluble and thus not toxic any more. Planktonic protists, diplonemids (Euglenozoa), are known for massive intracellular accumulations of barite, celestite (SrSO_4_) and strontiobarite (Ba,Sr)SO_4_ (Pilatová et al., 2023). The occurrence of barite is common in Zygnematophyceae, for example in desmids like *Closterium* and *Micrasterias denticulata* (e.g. Brook et al., 1980; Meindl, 1984; Wilcock et al., 1989; Krejci et al., 2011; Felhofer et al., 2021). In *Micrasterias* Raman bands at 986 cm^−1^, 615 cm^−1^, and 452 cm^−1^ were assigned to barite; exactly these three bands were reported as the strongest in barite (Zhou et al., 2020). The same bands (985 cm^−1^, 453 cm^−1^ and a smaller peak in the 615 cm^−1^ region) were also detected in the present study in living *Spirogyra* sp. ‘Greece’. In the present study barite crystals were observed in 2D SEM sections of *Spirogyra* sp. ‘Tyrol’ and *S. mirabilis* (**Figures 4D, E**) and TEM sections of *Spirogyra* sp. ‘Tyrol’ (**Figure 5C**) in ‘secondary vacuoles’ and compartments that resulted from degradation of chloroplasts. This is in contrast to the suggestion that barite crystals are formed in the cytoplasm of vegetative *Spirogyra* sp., as they are transported with the cytoplasmic streaming (Barbosa et al., 2022). Other than a contribution to barium detoxification (Niedermeier et al., 2018), the function of barite crystals in Zygnematophyceae remains largely unknown. Here we report on a large abundance of the barite crystal in ‘secondary vacuoles’ of mature zygospores (**Figure 4G**), suggesting a similar mechanism.

### Zygospore cell wall development is similar in all investigated strains

Previously, the zygospore cell wall development was investigated in *Spirogyra mirabilis* (Permann et al., 2021a) and the *Spirogyra* sp. ‘Tyrol’ strain (Permann et al., 2022) by TEM and Raman spectroscopy. The general architecture of the exo-, meso- and endospore in mature zygospores could be confirmed in the present study also for *Spirogyra* sp. ‘Greece’. In this strain also a young zygospore after conjugation was 3D reconstructed after SBF SEM, showing that only the thin exo- and endospore were present at this stage, while the mesospore developed later. This is in consensus with observations from developing *Zygnema vaginatum* zygospores (Permann et al., 2023). The thickness of the individual layers of the 3D reconstruction could furthermore be determined, which was above 4 µm in mature zygospores of *Spirogyra* sp. ‘Greece’. This is in the expected range for *Spirogyra* sp. zygospores (Stancheva et al., 2013). A punctate mesospore with a thickness of 3-6 µm has been described eg. for *S. australica*, while many investigated species had a smooth mesospore (Stancheva et al., 2013). In other literature, *Spirogyra* sp. zygospores with a reticulate-scrobiculate mesospore appearance were also described (Stancheva et al., 2013), but as stated above it was not the aim of the present study to perform a taxonomic treatment based on the morphological differences.

## Conclusion

In conclusion, this study is to the best of our knowledge the first study using 3D reconstruction of *Spirogyra* sp. zygospores to investigate their development and maturation process. With the SBF SEM technique we could get new insights into the quantitative changes of storage compounds like starch granules and LDs and the rearrangement of vacuoles and the quantitative increase in abundance of barite crystals from young to mature zygospores. Based on the morphological changes of the starch granules their amylose:amylopectin ratio should be determined in future studies, indicative of the accessibility to enzymatic digestion. Moreover, further information on the composition of LDs is needed, especially if they have the same composition in vegetative cells and zygospores. The biological function of the here observed massive barite accumulations is a further topic to be addressed. As we observed strain specific differences of the zygospore maturation process, it would also be rewarding to get insight if these changes are phylogenetically significant.

## Data availability statement

The raw data supporting the conclusions of this article will be made available by the authors, without undue reservation.

## Author contributions

SA: Formal analysis, Investigation, Methodology, Visualization, Writing – original draft. CP: Investigation, Writing – review & editing, Methodology, Resources. NX: Formal analysis, Methodology, Writing – review & editing. GT: Investigation, Methodology, Writing - review & editing. AH: Conceptualization, Funding acquisition, Investigation, Project administration, Supervision, Writing – original draft, Writing – review & editing.
