# Supplementary figures and images for: Zygospore development of Spirogyra (Charophyta) investigated by serial block-face scanning electron microscopy and 3D reconstructions
Source: Front Plant Sci. 2024 Mar 14;15:1358974. doi: 10.3389/fpls.2024.1358974 (PMC10978657; doi:10.3389/fpls.2024.1358974)

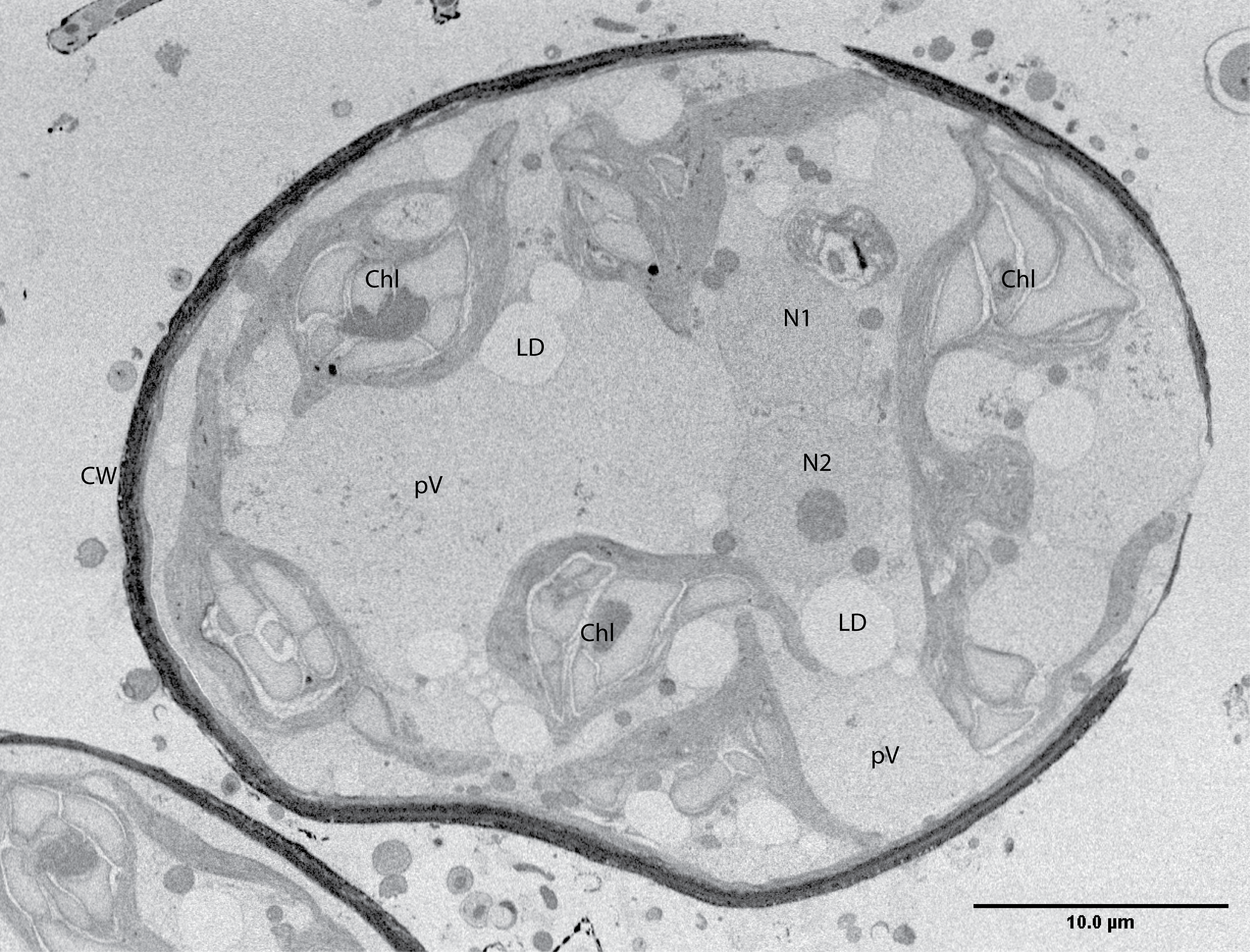

Supplement: Supplementary file 1 [file Image_1.tif]

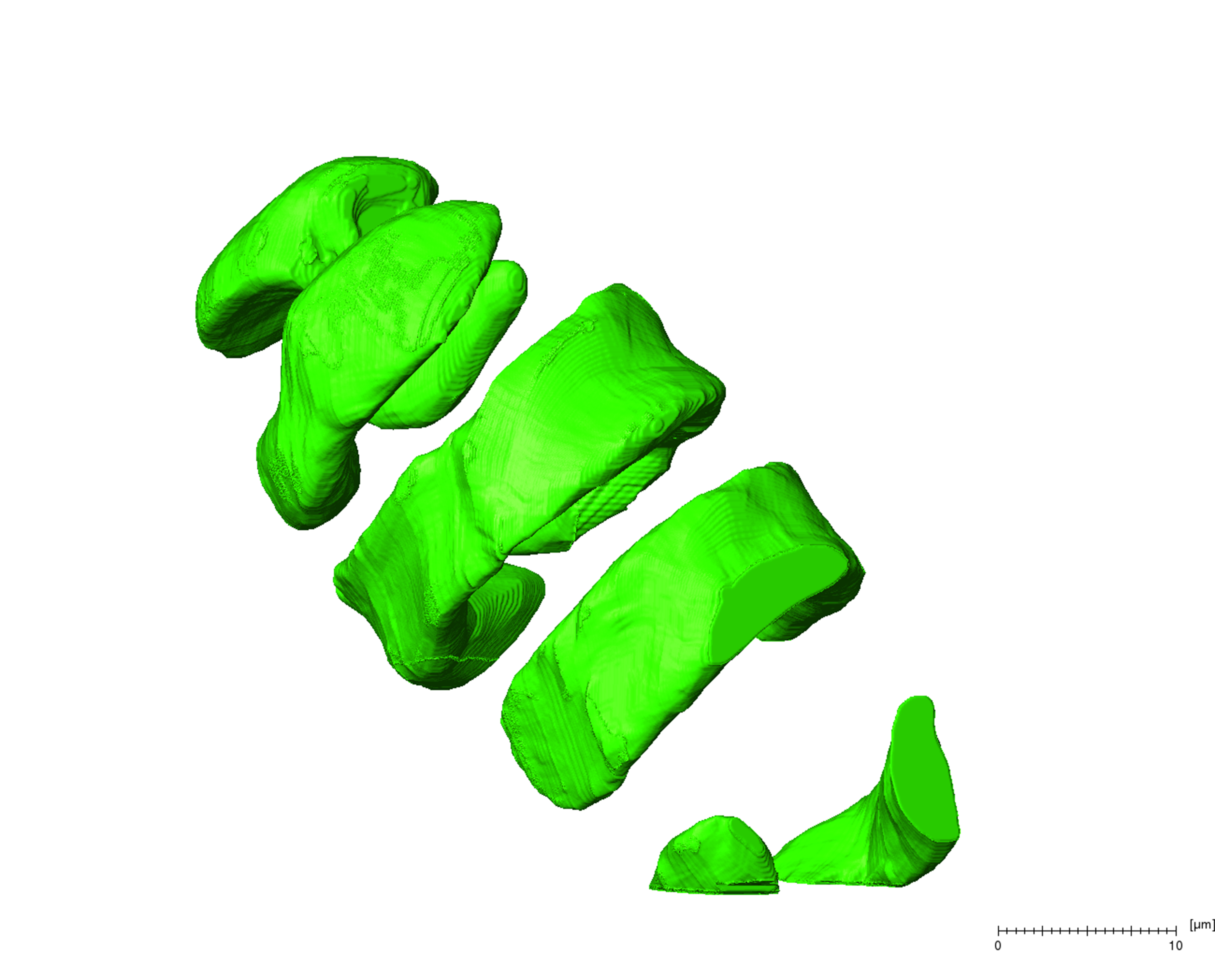

Supplement: Supplementary file 2 [file Image_2.tif]
